# Carbohydrate-Based Ice Recrystallization Inhibitors Increase Infectivity and Thermostability of Viral Vectors

**DOI:** 10.1038/srep05903

**Published:** 2014-07-31

**Authors:** Shahrokh M. Ghobadloo, Anna K. Balcerzak, Ana Gargaun, Darija Muharemagic, Gleb G. Mironov, Chantelle J. Capicciotti, Jennie G. Briard, Robert N. Ben, Maxim V. Berezovski

**Affiliations:** 1 Department of Chemistry, University of Ottawa, Ottawa, Ontario K1N 6N5, Canada

## Abstract

The inability of vaccines to retain sufficient thermostability has been an obstacle to global vaccination programs. To address this major limitation, we utilized carbohydrate-based ice recrystallization inhibitors (IRIs) to eliminate the cold chain and stabilize the potency of Vaccinia virus (VV), Vesicular Stomatitis virus (VSV) and Herpes virus-1 (HSV-1). The impact of these IRIs was tested on the potency of the viral vectors using a plaque forming unit assay following room temperature storage, cryopreservation with successive freeze-thaw cycles and lyophilization. Viral potency after storage with all three conditions demonstrated that *N*-octyl-gluconamide (NOGlc) recovered the infectivity of shelf stored VV, 5.6 Log_10_ PFU mL^−1^ during 40 days, and HSV-1, 2.7 Log_10_ PFU mL^−1^ during 9 days. Carbon-linked antifreeze glycoprotein analogue ornithine-glycine-glycine-galactose (OGG-Gal) increases the recovery of VV and VSV more than 1 Log_10_ PFU mL^−1^ after 10 freeze-thaw cycles. In VSV, cryostorage with OGG-Gal maintains high infectivity and reduces temperature-induced aggregation of viral particles by 2 times that of the control. In total, OGG-Gal and NOGlc preserve virus potency during cryostorage. Remarkably, NOGlc has potential to eliminate the cold chain and permit room temperature storage of viral vectors.

Vaccination is a public health success story saving 3 million lives each year. Many vaccines contain live attenuated viruses that have been cultivated under conditions that disable their virulent properties, or closely related but less dangerous viral vectors to produce a broad immune response or/and an anti-cancer (oncolytic) effect. The inability of viral vaccines to retain sufficient thermostability has been a significant obstacle to global vaccination programs and viral-based therapy^1^,^2^. Elevated temperatures damage live viruses and while cryopreservation is the preferred method of storage, freezing dramatically reduces the titer of the virus. Furthermore, exposure to low temperatures associated with cryopreservation results in virus agglomeration, rendering the vaccine ineffective^3^,^4^. The World Health Organization has mandated that, in order for a vaccine to be considered “effective”, less than 1 Log_10_ decrease in the original titer is tolerated^5^,^6^. Several approaches like biomineralization, addition of silk or albumin have been applied but unfortunately they often have multi-step preparation protocols or cause unwanted immune responses^3^,^4^,^5^,^7^,^8^,^9^,^10^,^11^,^12^,^13^. Consequently, novel methods for the preservation of vaccines are urgently required.

In this work, we examined the activity and thermostability of three live viral vectors from vesicular stomatitis virus (VSV), vaccinia virus (VV) and herpes simplex virus type 1 (HSV-1), all of which are popular candidates for cancer vaccine development. For example, VSV-Δ51, has been shown to possess potent oncolytic properties^14^ against a large number of potential tumor types^15^. VSV is a small bullet-shaped negative-strand RNA virus from the *Rhabdoviridae* family^16^. VSV selectively attacks tumor cells by taking advantage of defects to the interferon pathway^17^. Subsequently, VSV has been considered for clinical trials by Recombinant DNA Advisory Committee of NIH^18^,^19^. Furthermore, VSV is being developed as a vaccine shuttle for an array of viral pathogens, such as HIV-1^20^, Ebola virus^21^, hepatitis B^22^ and C^23^. JX-594 strain of VV is a member of the poxvirus family and has a large linear double-stranded DNA genome of approximately 200 kbp in length that encodes ~ 250 genes. It has several attributes that make it particularly well suited as an anticancer therapeutic^24^. VV is designed to attack cancer through three diverse mechanisms of action: 1) the lysis of cancer cells through viral replication, 2) the reduction of the blood supply to tumors through vascular targeting and destruction, and 3) the stimulation of the body's immune response against cancer cells. An attenuated HSV-1 strain, known as talimogene laherparepvec is a DNA oncolytic virus currently being studied for the treatment of melanoma and other advanced cancers by Amgen. With the announcement of positive results in March 2013, it is the first oncolytic virus to be proven effective in a Phase III clinical trial.

We examined thirteen compounds (all compounds can be found in Supporting Information) to preserve VV, HSV-1 and VSV at room temperature storage, during successive freeze-thaw cycles and lyophilization, and identified three promising ice recrystallization inhibitors, ornithine-glycine-glycine-galactose (OGG-Gal), *N*-octyl-d-gluconamide (NOGlc) and *N*-octyl-d-galactonamide (NOGal) (Figure 1a). All of these compounds demonstrate the ability to inhibit ice recrystallization^3^,^25^ and significantly increase the infectivity and thermostability of the viruses. OGG-Gal is a *C*-linked analogue of naturally occurring antifreeze glycoproteins (AFGPs)^26^ which allow arctic fish to survive in sub-zero temperatures^27^. OGG-Gal has been shown to be a potent inhibitor of ice recrystallization without the property of thermal hysteresis^26^, which can be detrimental to biological samples at low temperatures. Until recently, many structure-function studies of native AFGPs were only assessed for thermal hysteresis activity and not ice recrystallization activity^28^. However, more recent studies have explored the relationships between AFGP analogues and IRI activity^29^. The open-chain carbohydrates or alditols, NOGlc and NOGal, are hydrogelators that are capable of immobilizing water molecules into three-dimensional networks having morphologies of fibers^30^,^31^. Their ability to inhibit ice recrystallization was assessed using the standard “splat-cooling” assay^26^,^32^ in which ice crystal size was determined using photographs of frozen ice wafers after a 30 min annealing time at -6.4°C. The mean grain ice crystal size was determined using domain recognition software^33^ and compared to a phosphate buffered saline (PBS) solution as a control. As illustrated in Figure 1b, OGG-Gal is an extremely potent IRI at a concentration of 5.5 μM. In comparison, alditol NOGlc exhibits potent IRI activity only at 500 μM. Shortening the hydrophobic side chain in NOGlc results in a loss of IRI activity, indicating that the amphiphilic nature of these alditols is an essential property^34^. Interestingly, replacing the glucose alditol portion of NOGlc with a galactose alditol portion (NOGal) results in weak to moderate IRI activity, indicating that the stereochemical arrangement of the hydroxyl groups in the polyol component is an essential structural feature necessary for potent IRI activity as these two compounds only differ by one single stereocentre (indicated by boxes in Figure 1a). It is hypothesized that the mechanism by which these compounds inhibit ice recrystallization is though the disruption of the bulk water present between ice crystal boundaries^35^. As ice crystals grow, all solutes are excluded and the ice recrystallization inhibitors (IRIs) are concentrated at the interface of two ice crystals, where the interface consists of semi-ordered ice (quasi-liquid layer) separated by a layer of bulk water. It is thought that the hydration shell of the IRIs disrupts the ordering of bulk water therefore causing an increase in energy for the transfer of water molecules from bulk water to the ice lattice. Although the structure and molecular weights of OGG-Gal and the small IRIs is significantly different, the mechanism for ice recrystallization is thought to be the same. Further studies must be performed to determine the toxicity and immunogenicity of these compounds in animals.

The most potent compound resulting in stabilization of VV and HSV-1 at room temperature is NOGlc. VV and HSV-1 with and without 250 μM NOGlc were stored at 22°C and the infectivity of the viruses was determined counting plaque-forming units (PFU) in a cell-based assay. These data are represented by curves of logarithmic infectivity versus time (Figure 2). As a control, the infectivity of VV in PBS declined more than 1 Log_10_ PFU mL^−1^ in the first 8 days of storage and the ability to infect host cells was completely lost after 40 days. However, in the presence of NOGlc the infectivity decreased by only 0.2 Log_10_ PFU mL^−1^ after 40 days storage at room temperature (Figure 2a). These results were confirmed by viral quantitative capillary electrophoresis (viral qCE)^36^, where the intact virus particles were separated from free DNA present after virus degradation (Figure S1). VV degraded slower when incubated with NOGlc than with PBS as more VV peaks and less DNA was observed. We also tested the ability of NOGlc to stabilize HSV-1 at ambient temperature. While the reduction of untreated HSV-1 infectivity was 1 Log_10_ PFU mL^−1^ after one day, NOGlc treated HSV-1 lost only 0.05 Log_10_ PFU mL^−1^ of its infectivity on the first day and only 0.41 Log_10_ PFU mL^−1^ after 9 days, the maximum number of tested days for HSV-1 (Figure 2b).

While working with VV and VSV we found that even after multiple exposures to −20°C temperatures, the addition of carbohydrate-based IRIs prevented the damage caused by successive freezing and thawing. For instance, VV treated with OGG-Gal, NOGal and NOGlc lost 0.3, 1.3 and 0.5 Log_10_ PFU mL^−1^ respectively after 10 freeze-thaw cycles, compared with 1.7 Log_10_ PFU mL^−1^ lost in a control experiment with PBS (Figure 3a). Titration experiments demonstrate that the protective effect and ultimately the infectivity of VV was improved with increasing concentrations of NOGlc. The maximum protective effect with NOGal for VV was observed at 62.5 nM (Figure S2a and Figure S2b). The infectivity of VSV in the presence of OGG-Gal and NOGlc was almost 2.1 Log_10_ higher than a control after 10 freeze-thaw cycles (Figure 3b). Titration experiments revealed the maximum protection of VSV at 31 μM of NOGlc and 3 μM of OGG-Gal Figure S2d and S2c).

Regardless of how effective a vaccine may be in the laboratory, its commercial potential will be limited due to poor stability during storage and distribution. Lyophilization (freeze-drying) is a well-established technique used in the pharmaceutical industry for stabilizing high-cost, labile bio-products, such as vaccines. We examined the ability of our carbohydrate-based IRIs to preserve VV and VSV during the lyophilization process. As controls, we had tested 2% bovine serum albumin, fetal calf serum and glycerol as preservers just prior the test virus lyophilization. The recovery of the test viruses was the same as diluted viruses in PBS. Prior to lyophilization, VV infectivity was 6 Log_10_ PFU mL^−1^ but in the presence of OGG-Gal, NOGal, NOGlc and PBS VV infectivity was reduced by 0.68, 1.37, 1.06 and 2 Log_10_ PFU mL^−1^, respectively (Figure 4a). OGG-Gal showed the most promising result with only 0.68 Log_10_ PFU mL^−1^ reduction compared to 2 Log_10_ of PBS control. The infectivity of VSV in the presence of OGG-Gal, NOGal, NOGlc and PBS was reduced by 5–6 Log_10_ PFU mL^−1^ (Figure 4b). The question of why do these compounds protect viral vectors appears to be complex, as the same compounds provide protection with both storage methods. It is accepted that the majority of damage to bio-materials during cryopreservation is due to ice recrystallization that occurs during the storage and thawing cycles (assuming adequate dehydration during the rate controlled freezing process as described by Mazur's two stage hypothesis of cryoinjury). In lyophilization, samples are flash frozen and ice crystals are removed by sublimation. Under both conditions it seems reasonable that the ability of an ice recrystallization inhibitor to mitigate ice growth would be beneficial and ultimately protect against cryoinjury in both processes. However, another reason for decreased infectivity in cryopreserved and lyophilized samples is aggregation or agglomeration of virus particles. Consequently, we evaluated the impact of OGG-Gal on VSV aggregation after one freeze-thaw cycle using two different VSV expressing either yellow fluorescent protein (YPF) or red fluorescence protein (RFP). Galasso G. et al. by using electron microscopy showed that when a cell monolayer is infected with a low proportion of virus, one active viral particle can infect one cell and an aggregation several infectious particles lead only one cell infection^37^. An overlay of 1% Agarose restricts the spread of virus to neighbor cells. Hence, in this assay, a single virus with either RFP or YFP will infect a single host cell. In instances where aggregation of the virus particles occurs, multiple virus particles enter the cell and both red and yellow fluorescence is observed. After viral infection of cells with a mixture of two viruses, we detected fluorescent proteins and their localizations inside the cells (Figure 5). In the presence of OGG-Gal, the agglomeration of VSV dropped almost 2 times from 34 ± 3.8% to 16 ± 3.0% (Figure 5b). It is interesting to note that direct addition of the carbohydrate-based IRIs to a virus sample increases its infectivity by approximately 20% (Figure S3). These results suggest the compounds disperse virus aggregates and stabilize individual virus particles in the solution. The abilities of OGG-Gal, NOGal and NOGlc to stabilize and preserve three different viruses during cryopreservation, lyophilization and room temperature storage are summarized in Table 1.

Considering the structural differences between OGG-Gal, a high-molecular weight glycopeptide, and NOGlc, a small-molecule non-ionic surfactant, it is interesting to see very little difference in infectivity. Both compounds are equally effective at protecting against cryo-injury after ten successive freeze-thaw cycles. Furthermore, both of these compounds are most effective with VSV but less effective in VV. In contrast, NOGal is not effective (infectivity is identical to the PBS control). The IRI activity of small molecules is presented in Figure 1b. Ice recrystallization inhibition is defined as the prevention of the re-organization of smaller ice crystals into larger ice crystals. This would occur during the thawing phase in the freeze-thaw cycles. Both OGG-Gal and NOGlc are potent inhibitors of ice recrystallization. NOGlc is effective at 0.5 mM. Given the effectiveness of this compound to inhibit ice recrystallization, which is a significant cause of cellular damage during cryopreservation, it seems likely that NOGlc (and OGG-Gal) may directly inhibiting cryoinjury of the virus particle. In contrast, NOGal does not inhibit ice recrystallization (despite the fact that it differs from NOGlc by only one stereocentre) to any appreciable extent and fails to protect VV and VSV against the cryoinjury associated with successive freezing-thawing. This result is also consistent with the hypothesis that inhibiting ice recrystallization injury in the virus particle is beneficial. Ice crystals can damage a large virus more than a small virus. For cryopreservation, the best compounds are amphiliphilc molecules possessing a hydrophilic head group and a hydrophobic tail group, as seen in the alditol structure NOGlc.

Maintaining the stability of viral vectors is an obstacle to global vaccination programs and viral-based therapy^1^,^2^,^12^,^38^,^39^. Unfortunately, most vaccines lose 50% of their activity when constructed and stored for one hour at room temperature^6^,^9^,^38^. The transportation, storage, and use of them consequently present challenges, particularly in developing countries. Unlike former efforts, direct addition of NOGlc is easy, economical and robust. Our results show that NOGlc protects significantly VV and HSV-1 infectivity at room temperature for days (Figure 2).

In conclusion, according WHO requirement for vaccines cold chain management, NOGlc increases the shelf life of VV from four days to more than forty days and HSV-1 from a day to more than nine days. NOGlc has opportunity to eliminate the cold chain from viral vector vaccine management.

## Author Contributions

**Figure 1:**
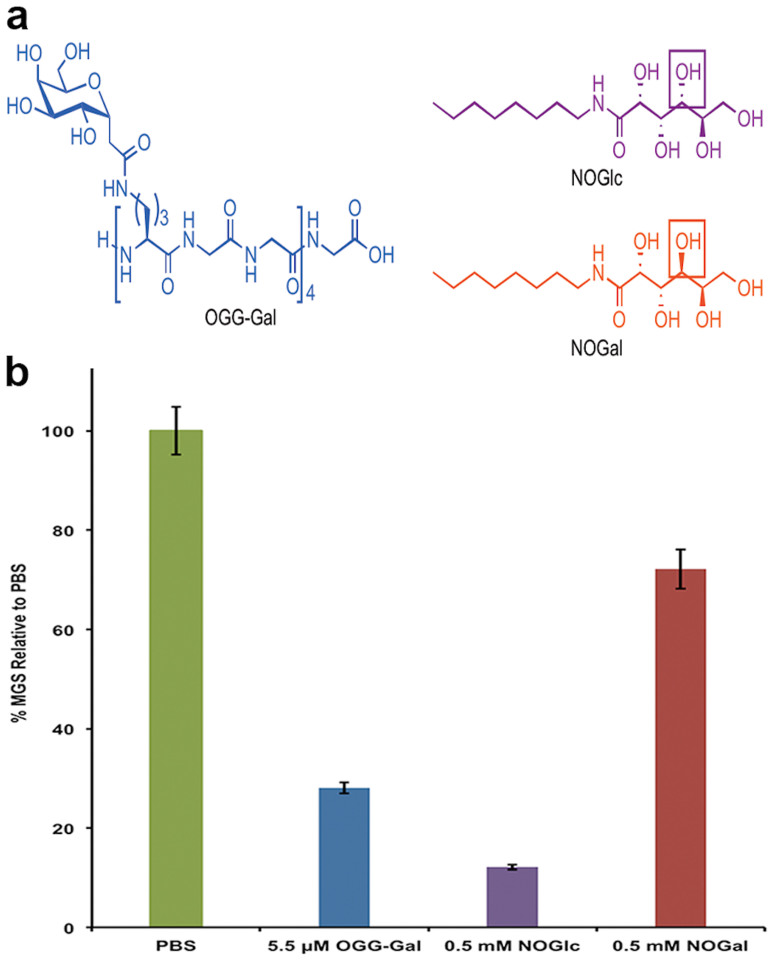
(a) Structures of ice recrystallization inhibitors OGG-Gal, NOGlc and NOGal. Boxes highlight differences in stereochemistry. (b) Ice recrystallization inhibition activity. The mean grain ice crystal sizes (MGS) are compared to a PBS standard. Error bars indicate standard error of the mean.

S.M.G. and M.V.B. conceived the idea and designed the research. S.M.G. performed experiments with viruses. A.K.B., C.J.C. and J.G.B. synthesized carbohydrate-based ice recrystallization inhibitors and prepared figure 1. A.G. and D.M. performed capillary electrophoresis experiments. G.G.M. performed mass spectrometry experiments to study virus-small molecule binding. S.M.G. and A.K.B. wrote the paper. R.N.B. and M.V.B. edited the manuscript and supervised the study. All authors read the manuscript, provided comments, and approved its content.

## Supplementary Material

Supplementary Information Supplementary Info

## Figures and Tables

**Figure 2:**
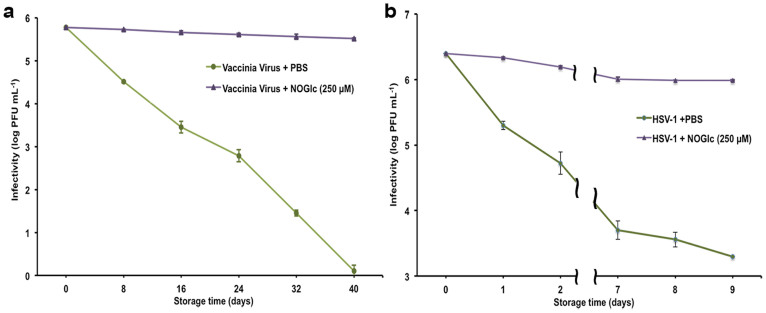
NOGlc impact on stored virus at 22°C: (a) VV infectivity during 40 days. (b) HSV-1 infectivity during 9 days. Error bars indicate standard error of the mean.

**Figure 3:**
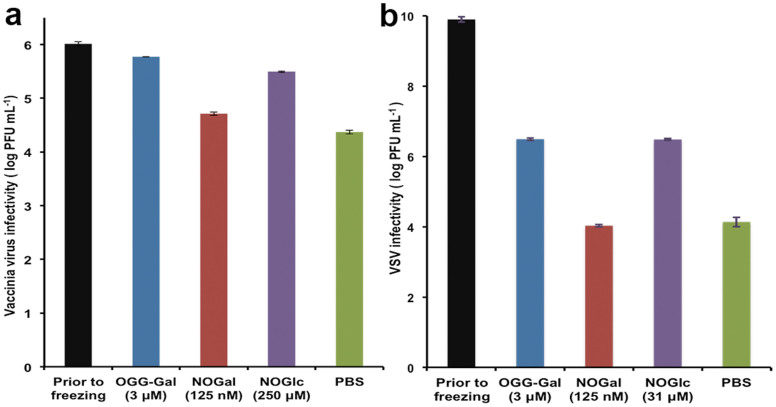
Impact of ice recrystallization inhibitors on viral vector infectivity after 10 freeze-thaw cycles on (a) VV and (b) VSV. Error bars indicate standard error of the mean.

**Figure 4:**
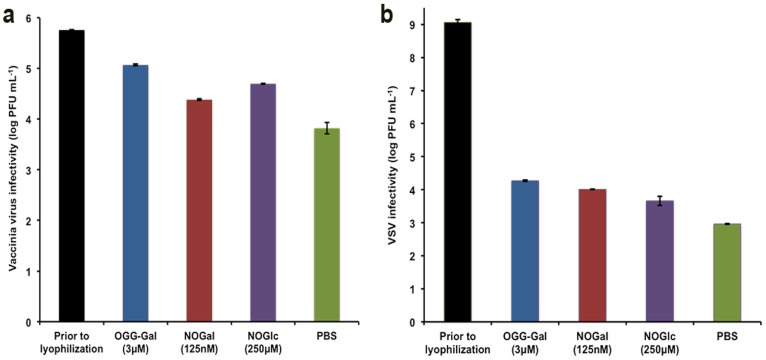
Effect of ice recrystallization inhibitors on viral vectors infectivity after lyophilisation of (a) VV and (b) VSV. Error bars indicate standard error of the mean.

**Figure 5:**
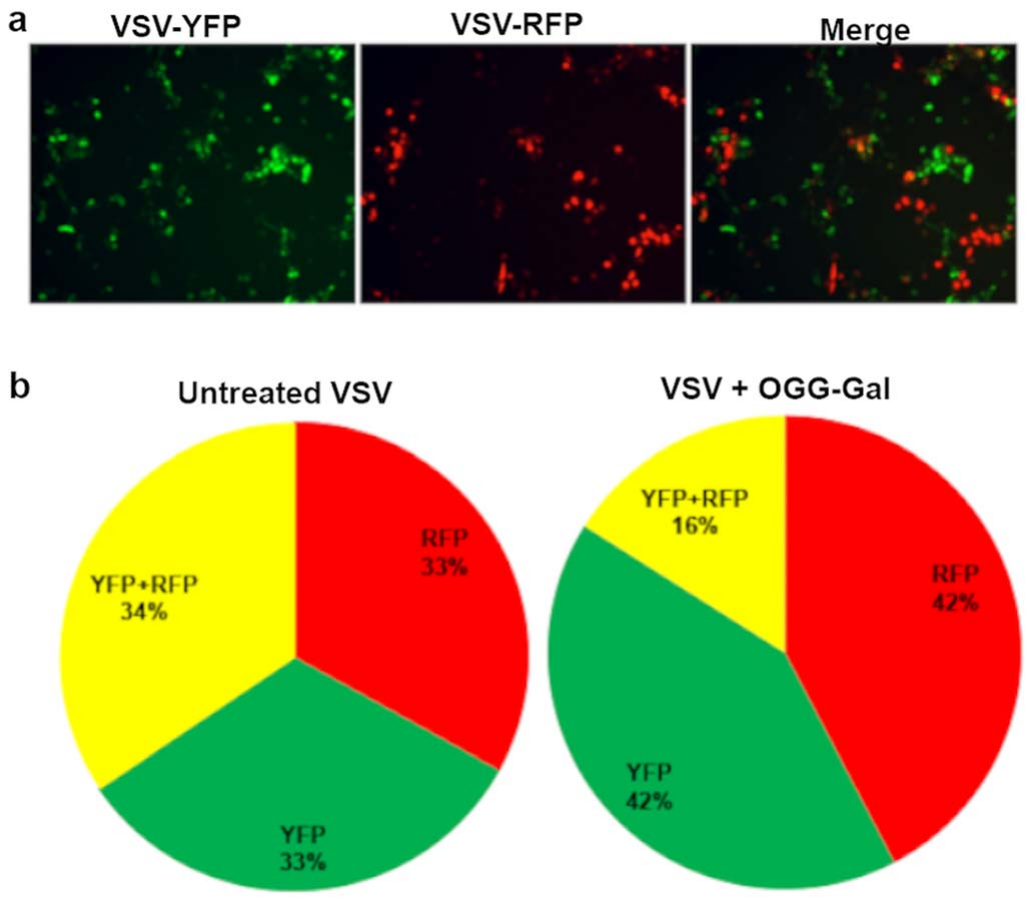
Cells infected with a mixture consisting of equal amounts of VSVs expressing YFP and RFP; (a) cells expressing YFP, RFP and both YFP and RFP, respectively. (b) Percentage of cells infected by YFP, RFP, and both YFP and RFP for untreated, and OGG-Gal treated VSV.

**Table 1:** The capacity of OGG-Gal, NOGal and NOGlc to recover three different viruses throughout cryopreservation, lyophilization and room temperature storage

		OGG-Gal	NOGal	NOGlc
Room Temperature	VV^1^	NS	NS	↑↑↑↑↑↑↑↑↑↑↑
	VSV^2^	NS	NS	NS
	HSV-1^3^	NS	NS	↑↑↑↑↑
Freeze-Thaw	VV^4^	↑↑	↑	↑↑
	VSV^4^	↑↑↑↑	NS	↑↑↑↑
	HSV-1^4^	NS	NS	NS
Lyophilization	VV	↑↑	↑	↑↑
	VSV	↑↑	↑↑	↑
	HSV-1	NS	NS	NS

↑ - represents recovered infectivity in 0.5 Log_10_ PFU mL^−1^ compare with PBS control.

NS - no significant effect was observed.

^1^after 40 days at RT.

^2^after 5 days at RT.

^3^after 9 days at RT.

^4^after 10 freeze-thaw cycles.
