# Supplementary material for: Carbohydrate-Based Ice Recrystallization Inhibitors Increase Infectivity and Thermostability of Viral Vectors
Source: Sci Rep. 2014 Jul 31;4:5903. doi: 10.1038/srep05903 (PMC4116624; doi:10.1038/srep05903)
Supplement: Supplementary Information — Supplementary Info [file srep05903-s1.doc]

**Supporting Information**

**Carbohydrate-Based Ice Recrystallization Inhibitors Increase Infectivity and Thermostability of Viral Vectors**

Shahrokh M. Ghobadloo, Anna K. Balcerzak, Ana Gargaun, Darija Muharemagic, Gleb G. Mironov, Chantelle J. Capicciotti, Jennie G. Briard, Robert N. Ben^*^, and Maxim V. Berezovski^*^

Department of Chemistry, University of Ottawa, Ottawa, Ontario K1N 6N5, Canada; University of Ottawa, 501 Smyth Road, Ottawa, Ontario K1H 8L6, Canada.

Corresponding authors:

Maxim V. Berezovski (maxim.berezovski@uottawa.ca)

Robert N. Ben (rben@uottawa.ca)

Table of Contents

1. Compounds [2](#__RefHeading___Toc253077228)

2. Biological Experimental Details [2](#__RefHeading___Toc253077229)

3. Figures [4](#__RefHeading___Toc253077230)

4. General Synthetic Experimental [8](#__RefHeading___Toc253077231)

5. Assessing Ice Recrystallization Inhibition (IRI) Activity [8](#__RefHeading___Toc253077232)

6. Synthetic Experimental Data [9](#__RefHeading___Toc253077233)

6.1. Synthesis of OGG-Gal [9](#__RefHeading___Toc253077234)

6.2. Synthesis of *N*-octyl-d-gluconamide (NOGlc) [13](#__RefHeading___Toc253077235)

6.3. Synthesis of *N*-octyl-d-galactonamide (NOGal) [13](#__RefHeading___Toc253077236)

7. Spectroscopic Data [14](#__RefHeading___Toc253077237)

8. References [22](#__RefHeading___Toc253077238)

# Compounds

The following compounds were assessed for their ability to preserve viral vectors:

Ornithine–glycine–glycine-galactose (OGG-Gal), *N*-octyl-d-galactonamide (NOGal), *N*-octyl-d-gluconamide (NOGlc), *N*-octyl-d-galactonamide (NOGal), *N*-butyl-gluconamide, β-octyl-galactopyranoside, *N*-methyl-*N*-octyl gluconamide, sodium *N*^α^-hexanoyl-*N*^ε^-decanoyl-l-lysinate, sodium *N*^α^,*N*^ε^-bis(hexanoyl)-l-lysinate, sodium *N*^ε^-dodecanoy-l-lysinate, *N*^α^-hexanoyl-*N*^ε^-hexyl-l-lysinate, *N*-octyl-β-d-galctopyranoside, l-lysine decyl ester dihydrochloride, l-lysine tetradecyl ester dihydrochloride, and l-lysine tetradecyl ester dihdrochloride.

# Biological Experimental Details

**Cells**

U2OS and Vero cells were grown in Dulbecco's Modified Eagle Medium (DMEM) supplemented with 10% fetal bovine serum (FBS).

**VSV Propagation**

VSV, a recombinant VSV expressing the yellow fluorescent protein (YFP) or red fluorescent protein (RFP) was obtained from Dr. John Bell’s laboratory. VSV was propagated on Vero cells in the presence of 10% FBS. At 24 h post infection, the cell culture supernatant was collected, and the cell debris was removed by centrifugation at 3,000 g for 20 min at 4 ºC. The virus was aliquoted and stored at -80 ºC after titration.

**VSV Titration**

The VSV plaque forming units were determined on a monolayer of Vero cells grown in 12- well culture plates. The cells were inoculated in duplicates of serial virus dilutions for 60 min at 37 ºC. Thereafter, Dulbecco’s Modified Eagle Medium (DMEM, Invitrogen, CA) with 1% low melting agarose was added to each well and the cells were incubated at 37 ºC. The cells were surveyed 24 h after infection using an Alfa Innotech Imaging System, Version 3.0.3.0 and the infectious virus titers were calculated based on the number of YFP- or RFP positive plaques per well and expressed as focus-forming units per milliliter (PFU mL-1).

**HSV-1 Titration**

A sample of serial diluted HSV-1 expressing GFP in 250 μL of serum-free medium was added to a Vero cell monolayer (0.4 × 10^6^ cells per well) in a 12-well culture plate. Inoculated cells were incubated for 1 h at 37 °C in a 5% CO2 humidified incubator and then cells were overlaid with 1 mL of fresh 1% agarose liquefied in DMEM. After 24 h of incubation, infected cells were visualized using Alfa Innotech Imaging System, Version 3.0.3.0 for GFP fluorescence. In addition, a standard plaque assay was achieved, where the same plates were fixed with methanol−acetic acid fixative (3:1 ratio), stained with Coomassie Brilliant Blue R solution, and white plaques were counted.

**VV propagation**

VV was propagated in U2OS monolayer cultures at 37°C. Cells were infected with VV for 1 hour in DMEM at 37°C.The media was then replaced with fresh DMEM containing 10% FBS. After 48 hours, the supernatant of the infected cells was harvested by centrifugation at 650 g for 30 minutes at 4 °C. The pellets were then suspended in DMEM and the virus was subsequently used as the stock of vaccinia.

**VV titration**

The serial dilutions of VV were added to U2OS cells maintained in 12-well plates. Following virus binding to the cells at 37◦C for 1 hour, virus inoculum was aspirated and an overlay solution (mixture of 1:1 volumes of 3% carboxymethylcellulose: 2x DMEM, 20% FBS) was added. After 48 hours of incubation, cells positive for GFP fluorescence were visualized by using Alfa Innotech Imaging System, Version 3.0.3.0. In addition, a standard plaque assay was performed, where the overlay was removed and the cell monolayer was stained with crystal violet for enumeration of the virus plaques.

**VSV aggregation test**

Equimolar concentrations of YFP and RFP-expressing VSV were incubated with and without the compound for 1 hour at 37 °C and then used to infect cells plated on a chamber slide. After 1 hour of infection, cells were washed and overlaid with 0.5% low melting agarose with DMEM and supplemented with 10% FBS. After 24 hours of incubation at 37 °C in a 5% CO2 humidified incubator, cells were washed and analysed by fluorescence microscopy. The cells expressing YFP, RFP, or both YFP and RFP were counted and analysed.

**VV incubation with NOGlc at 22 °C and analysis by capillary electrophoresis**

Two aliquots of VV were prepared, with PBS, and with 40.67 µM NOGlc, and incubated at room temperature for 12 days. Prior to separating each sample by capillary electrophoresis, they were stained with 2.0 µM YOYO-1 fluorescent nucleic acid dye (Invitrogen, CA). As a control, a sample from the two aliquots of VV was stained with 2.0 µM YOYO-1 dye and subjected to separation by capillary electrophoresis without any incubation (Day 0).

A ProteomeLab PA 800 capillary electrophoresis system from Beckman-Coulter, Brea, USA, was used to separate VV. Fluorescence was induced by a 488-nm Ar-ion laser and detected at 520±10 nm. A bare silica-fused capillary was used, 60 cm in total length with 50 cm from injection to the detection point, an outer diameter of 365 µm and an inner diameter of 75 µm. The injections were done by a pressure pulse with hydrodynamic injection volumes of 39 nL. The electric field during the separation was 250 V cm^-1^, with the positive charge at the inlet and ground at the outlet. The capillary temperature was maintained at 15 °C for the duration of the experiment. The run buffer was 25 mM sodium tetraborate at pH 9.84. Prior to each injection, the capillary was rinsed by applying 20.0 psi of 100 mM HCl, 100 mM NaOH, ddH_2_O, and 25 mM Borax for 2.5 minutes each. 32 KaratTM software (Beckman-Coulter, Brea, USA) was used for recording the electropherograms and final electropherograms were produced by Excel (Microsoft).

**Air-dried VV and VSV infectivity assay**

VV or VSV suspended in DMEM with or without the selected compounds was added to flat-bottom 12-well culture dishes. The suspension was directly added to the polystyrene bottom of the wells. The virus was dried under a laminar flow for 60 minutes before the plate was covered with a lid and kept at room temperature. The dried virus was resuspended in DMEM and titrated on Vero cells. The infectivity of VV (600,000 PFU mL^−1^) suspended in selected IRIs, which were dried under a laminar flow for 60 min before being covered with a lid and kept at room temperature, are depicted in (**Figure S4b**). The results of the plaque-forming assay show that VV in the presence of NOGlc was twice as infective as the control. In the same way to determine the effectiveness of IRIs at stabilizing air-dried VSV, a solution of OGG-Gal with VSV (4×10^10^ PFU mL^-1^) was dried under laminar flow for 60 min at room temperature. The assay indicated that OGG-Gal is 30% more effective at protecting VSV than the control (**Figure S4b**).

# Figures


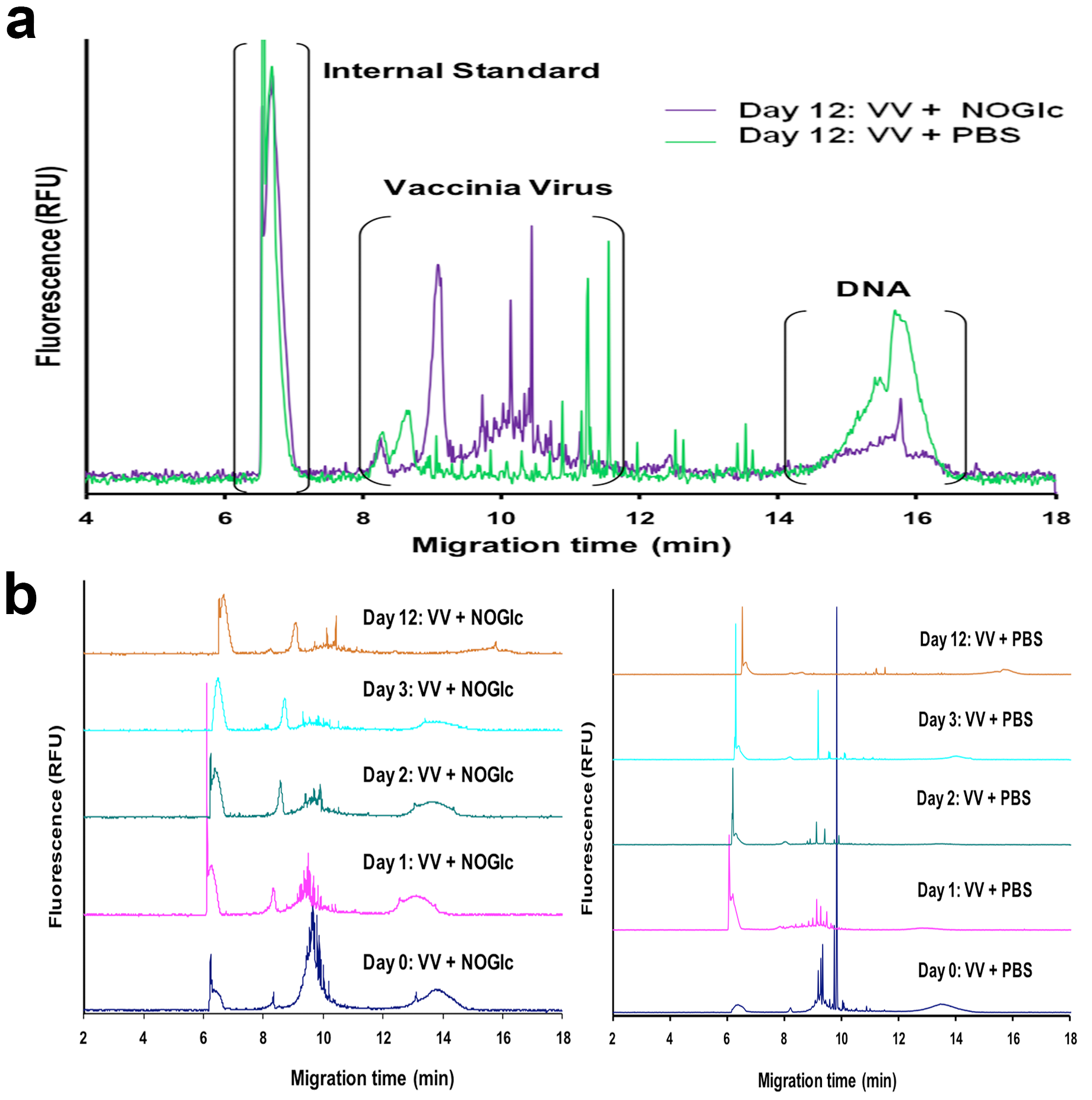


**Figure S1**. Electropherograms for VV separated by capillary electrophoresis (CE) and detected by laser induced fluorescence after incubation with PBS or NOGlc at 22 °C for 12 days. A) VV separated by CE after 12 days of incubation with NOGlc and PBS. B) Separation of VV by CE on various days of incubation with NOGlc and PBS. All samples were stained with YOYO®-1 dye. CE separations were performed in a 60 cm long capillary under 250 V cm^-1^ in 25 mM borax buffer at 15 °C


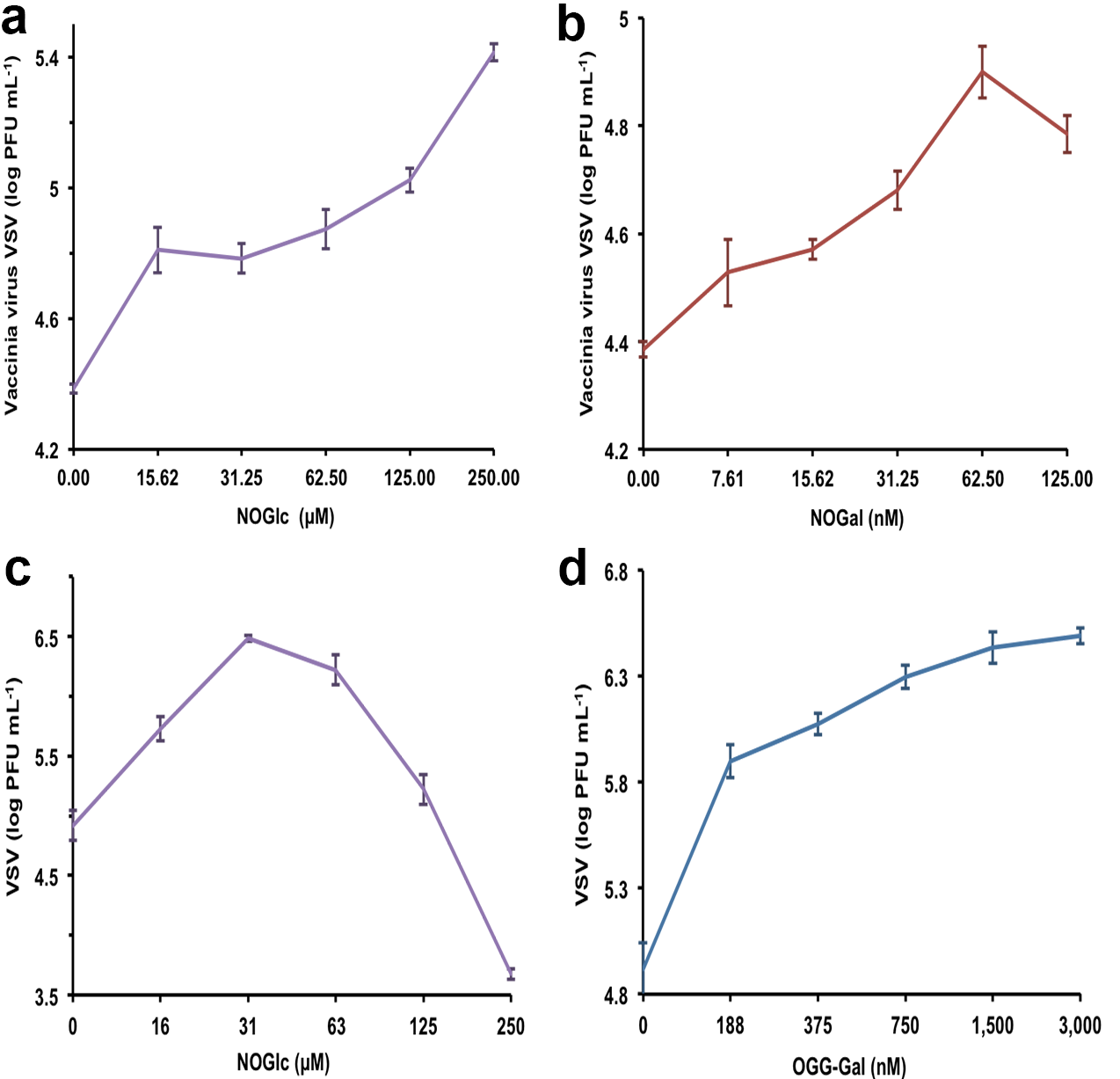


**Figure S2.** Dosage effect assessment by number of plaques formed. a) VV treated with NOGlc b) VV treated with NOGal c) VSV treated with NOGlc d) VSV with OGG-Gal


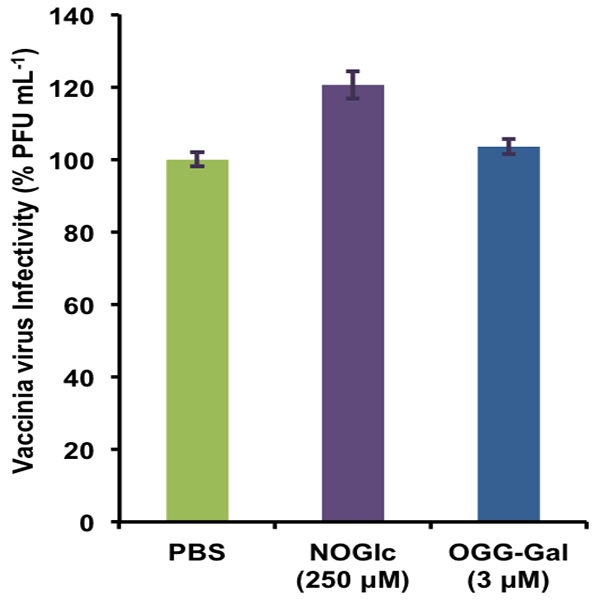


**Figure S3.** VV infectivity after 1 h incubation in NOGlc and OGG-Gal solution and PBS control


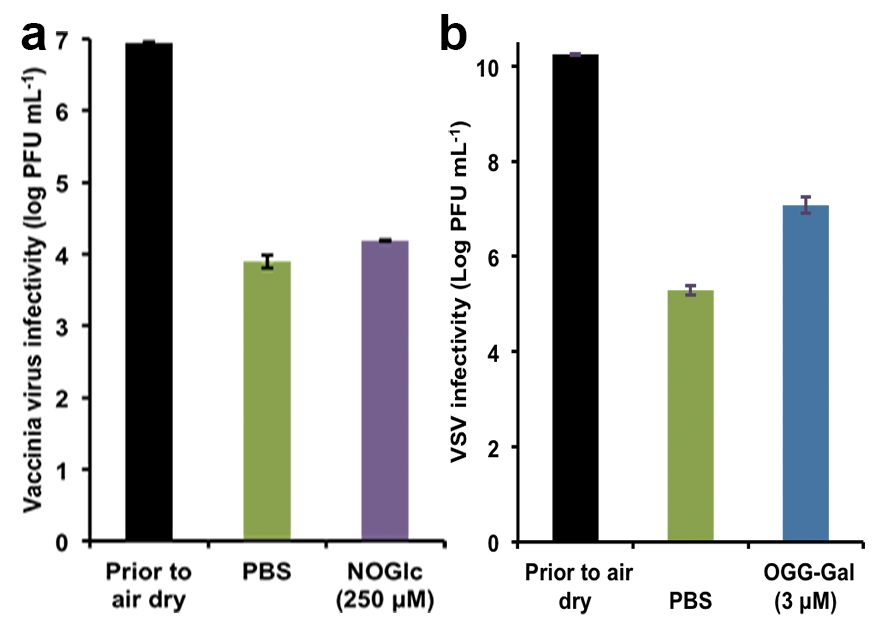


**Figure S4.** Air-dried viral vectors infectivity. a) air-dried VV in NOGlc solution b)VSV in OGG-Gal solution.

# General Synthetic Experimental

All anhydrous reactions were performed in flame-dried or oven-dried glassware under a positive pressure of dry argon or nitrogen. Air or moisture-sensitive reagents and anhydrous solvents were transferred with oven-dried syringes or cannulae. All flash chromatography was performed with EMD Silica gel 60 (230-400 mesh). All solution phase reactions were monitored using analytical thin layer chromatography (TLC) with 0.2 mm pre-coated silica gel aluminum plates 60 F254 (E. Merck). Components were visualized by illumination with a short-wavelength (254 nm) ultraviolet light and/or staining (ceric ammonium molybdate, potassium permanganate, or phosphomolybdate stain solution).

All solvents used for anhydrous reactions were distilled. Tetrahydrofuran (THF) and diethyl ether were distilled from sodium/benzophenone under nitrogen. Dichloromethane, acetonitrile, triethylamine, benzene and diisopropylethylamine (DIPEA) were distilled from calcium hydride. *N,N*-dimethylformamide (DMF) was stored over activated 4Å molecular sieves under argon.

^1^H (300, 400, or 500 MHz) and ^13^C NMR (75, 100 or 125 MHz) spectra were recorded at ambient temperature on a Bruker Avance 300, Bruker AM 360, Bruker Avance 400 or Bruker Avance 500 spectrometer. Deuterated chloroform (CDCl_3_), methanol (CD_3_OD), or deuterium oxide (D_2_O) were used as NMR solvents, unless otherwise stated. Chemical shifts are reported in ppm downfield from TMS and corrected using the solvent residual peak or TMS as an internal standard. Splitting patterns are designated as follows: s, singlet; d, doublet; t, triplet; q, quartet; m, multiplet and br, broad. Low resolution mass spectrometry (LRMS) was performed on a Micromass Quatro-LC Electrospray spectrometer with a pump rate of 20 μL/min using electrospray ionization (ESI) or a Voyager DE-Pro matrix-assisted desorption ionization-time of flight (MALDI-TOF), (Applied Biosystem, Foster City, CA) mass spectrometer operated in the reflectron/positive-ion mode with DHB in 20% EtOH/H_2_O as the MALDI matrix. High resolution mass spectrometry (HRMS) data was acquired on Applied Biosystems/Sciex QStar (Concord, ON). Samples in CH_2_Cl_2_/MeOH 1:1 were mixed with Agilent ES tuning mix for internal calibration, and infused into the mass spectrometer at 5 μL/min.

# Assessing Ice Recrystallization Inhibition (IRI) Activity

Sample analysis for IRI activity was performed using the “splat cooling” method as previously described. In this method, the analyte was dissolved in phosphate buffered saline (PBS) solution and a 10 *μL* droplet of this solution was dropped from a micropipette through a two meter high plastic tube (10 cm in diameter) onto a block of polished aluminum precooled to approximately -80 °C. The droplet froze instantly on the polished aluminum block and was approximately 1 cm in diameter and 20 *μm* thick. This wafer was then carefully removed from the surface of the block and transferred to a cryostage held at -6.4 °C for annealing. After a period of 30 min, the wafer was photographed between crossed polarizing filters using a digital camera (Nikon CoolPix 5000) fitted to the microscope. A total of three images were taken from each wafer. During flash freezing, ice crystals spontaneously nucleated from the supercooled solution. These initial crystals were relatively homogeneous in size and quite small. During the annealing cycle, recrystallization occurred, resulting in a dramatic increase in ice crystal size. A quantitative measure of the difference in recrystallization inhibition of two compounds X and Y is the difference in the dynamics of the ice crystal size distribution. Image analysis of the ice wafers was performed using a novel domain recognition software (DRS) program. This processing employed the Microsoft Windows Graphical User Interface to allow a user to visually demarcate and store the vertices of ice domains in a digital micrograph. The data was then used to calculate the domain areas. All data was plotted and analyzed using Microsoft Excel. The mean grain (or ice crystal) size (MGS) of the sample was compared to the MGS of the control PBS solution for that same day of testing. IRI activity is reported as the percentage of the MGS (% MGS) relative to the PBS control, and the % MGS for each sample was plotted along with its standard error of the mean. Large percentages represent a large MGS, which is indicative of poor IRI activity.

# Synthetic Experimental Data

## Synthesis of OGG-Gal

Scheme S1. Synthesis of galactopyranosyl acid 4

**2,3,4,6-tetra-*O*-acetyl-α-d-galactopyranosyl bromide (2)**

To commercially available d-pyranose pentaacetate (**1**, 5 g, 12.81 mmol), 30 mL of HBr in AcOH (33% solution) was added at room temperature. The reaction was stirred for 40 minutes and then diluted with CH_2_Cl_2_. The solution was transferred to a separatory funnel containing ice and the organic layer was washed with ice water until neutral pH. The organic extract was dried over MgSO_4_, filtered and concentrated to afford product in 89% yield. The crude product was crystallized from diethyl ether to form a white powder.

**^1^H NMR** (360 MHz, CDCl_3_) δ 6.56 (1H, d, *J=*3.9 Hz), 5.39 (1H, m), 5.22 (1H, dd, *J=*4.2, 8.2 Hz), 4.85 (1H, d, *J=*4.3 Hz), 4.82 (1H, d, *J=*4.2 Hz), 4.25 (1H, m), 3.97 (2H, m), 1.98 (3H, s), 1.98 (3H, s), 1.87 (3H, s), 1.82 (3H, s); **^13^C NMR** (90 MHz, CDCl_3_) δ 169.98, 169.70, 169.64, 169.42, 88.19, 70.94, 67.73, 67.51, 66.78, 60.67, 20.47, 20.37, 20.31; LRMS (ESI): Calcd for C_14_H_23_BrNO_9_ [M+NH_4_]^+^ 429.2, found 429.9.

**Allyl 2,3,4,6-tetra-*O*-acetyl-α-d-galactopyranoside (3)**

To a solution of 2,3,4,6-tetra-*O*-acetyl-α-d-galactopyranosyl bromide (**2**, 1.24 g, 3.01 mmol) in benzene (15 mL), allyl phenyl sulfone (1.418 mL, 7.52 mmol), and bis-tributyl tin (2.19 mL, 4.21 mmol) were added. The solution was degassed and sonicated for 30 minutes under argon atmosphere; the sealed flask was irradiated for 9 hours under a 450W mercury arc lamp. The reaction was monitored using TLC at time intervals of 2, 6 and 8 hours. The reaction mixture was loaded directly onto a silica gel column packed with hexanes. The organostannanes were flushed with 3 void volumes of hexanes, after which the solvent system was changed to 5:1 hexanes/EtOAc to remove the unreacted allyl phenyl sulfone. The product was eluted with 3:1 hexanes/EtOAc and concentrated to afford the allylated derivative as colorless oil (88% yield).

**^1^H NMR** (360 MHz, CDCl_3_) δ 5.76 (1H, dddd, J=6.5, 7.1, 10.0, 16.6 Hz), 5.42 (1H, dd, J=2.4, 3.1 Hz), 5.28 (1H, dd, J=4.8, 9.3 Hz), 5.22 (1H, dd, J=3.1, 9.3 Hz), 5.13 (1H, ddd, J=1.6, 3.0, 16.6 Hz), 5.12 (1H, ddd, J=1.4, 3.0, 10.0 Hz), 4.30 (1H, ddd, J=4.7, 5.2, 10.3 Hz), 4.21 (1H, dd, J=8.9, 12.5 Hz), 4.09 (2H, m), 2.46 (1H, m), 2.29 (1H, m), 2.12 (1H, s), 2.07 (1H, s), 2.04 (1H, s), 2.03 (1H, s); **^13^C NMR** (90 MHz, CDCl_3_) δ 170.5, 170.1, 169.9, 169.8, 133.3, 117.6, 71.4, 68.2, 67.9, 61.4, 30.9, 20.8, 20.7, 20.6; LRMS (ESI): Calcd for C_17_H_25_O_9_ [M+H]^+^ 373.4, found 373.1.

**2,3,4,6-tetra-*O*-acetyl-α-d-galactopyranosyl acid (4)**

Allyl 2,3,4,6-tetra-*O*-acetyl-α-d-galactopyranoside (**3**, 0.83 g, 2.23 mmol) was dissolved in 14 mL of a solution of (2:2:3) acetonitrile: carbon tetrachloride: water, followed by the addition of sodium periodate (1.90 g. 8.92 mmol). A catalytic amount of ruthenium trichloride trihydrate was then added, and the reaction was allowed to stir at room temperature for 2-3 hours. The solution was filtered through celite and transferred to a separatory funnel with dichlroromethane. The organic layer was washed successively with saturated ammonium chloride solution, saturated brine solution, dried over MgSO_4_, filtered and concentrated to afford the carboxylic acid derivative in 85 % yield.

**^1^H NMR** (360 MHz, CDCl_3_) δ 5.43 (1H, t, *J*=2.8 Hz), 5.33 (1H, dd, *J*=8.9, 5.0 Hz,), 5.17 (1H, dd, *J* =8.9, 3.3 Hz), 4.70 (1H, ddd, *J*=9.3, 8.3, 5.3 Hz), 4.30-4.08 (3H, m), 2.73 (1H, dd, *J*=15.6, 8.7 Hz), 2.63 (1H, dd, *J*=15.6, 5.7 Hz), 2.13 (3H, s), 2.07 (3H, s), 2.04 (6H, s); **^13^C NMR** (90 MHz, CDCl_3_) δ 175.5, 170.8, 170.1, 170.0, 169.7, 69.4, 68.9, 67.8, 67.6, 67.1, 61.2, 33.15, 20.7; IR (thin film): 3706-2355, 1748 cm^-1^.

**Benzyl Fluorenylmethoxycarbonyl-l-ornithine-1-(2,3,4,6-tetra-*O*-acetyl-α-d-galacto-pyranoside) (6)**

To a solution of fully protected amino acid derivative **5** (0.21 g, 0.38 mmol) in 10 mL of CH_2_Cl_2_, 2 mL of TFA was added. The reaction was stirred for 40 minutes and then concentrated under reduced pressure. The syrup was re-dissolved in a 1:1 mixture of toluene and CH_2_Cl_2_, concentrated, then re-dissolved in diethyl ether and concentrated to afford a white solid. To a solution of carboxylic acid carbohydrate derivative (0.15 g, 0.38 mmol), HBTU (0.17 g, 0.46 mmol) in 15 mL of CH_2_Cl_2_, and 0.20 mL (0.15 g, 1.14 mmol) of DIPEA were added. The reaction was allowed to stir for 20 minutes and then the deprotected amino acid derivative was added. After stirring overnight, the reaction mixture was washed successively with saturated ammonium chloride, water, brine, dried over MgSO_4_ and concentrated. The product was purified by flash column chromatography in 50:1 CH_2_Cl_2_ and MeOH to afford a *C*-linked glycosyl amide building block in 83% yield.

**^1^H NMR** (300 MHz, CDCl_3_) δ 7.76 (2H, d, *J*=7.5 Hz), 7.59 (2H, d, *J*=7.21 Hz), 7.42-7.28 (9H, m), 6.12 (1H, br s), 5.55 (1H, d, *J*=8.4 Hz), 5.4 (1H, t, *J*=3.3 Hz), 5.29-5.23 (1H, m), 5.17-5.12 (3H, m), 4.67-4.61 (1H, m), 4.46-4.34 (3H, m), 4.25-4.19 (2H, m), 4.15-4.09 (2H, m), 3.29-3.23 (2H, m), 2.57-2.47 (1H, m), 2.41-2.34 (1H, m), 2.10 (3H, s), 2.05 (3H, s), 2.04 (3H, s), 2.00 (3H, s), 1.93-1.82 (1H, m), 1.73-1.47 (3H, m); **^13^C NMR** (75 MHz, CDCl_3_) δ 171.9, 170.5, 169.9, 169.7, 169.5, 169.2, 156.0, 143.6, 141.2, 135.0, 128.6, 128.5, 127.7, 127.0, 125.0, 119.9, 69.4, 68.7, 67.8, 67.7, 67.2, 67.0, 66.8, 61.0, 53.5, 47.0, 38.6, 34.4, 30.0, 25.3, 20.7, 20.6; LRMS (ESI): Calcd for C_43_H_49_N_2_O_14_ [M+H] ^+^ 817.8, found 817.4.

**Fluorenylmethoxycarbonyl-l-ornithine-1-(2,3,4,6-tetra-*O*-acetyl-α-d-galacto-pyranoside) (7)**

To a solution of benzyl ester **6** in 1:1 ethanol/ethyl acetate, a catalytic amount (8% w/w) of palladium on charcoal (10% w/w) was added. The reaction mixture was stirred under H_2_ atmosphere and monitored via TLC. After the reaction was completed, the catalyst was gravity filtered through Whatman No.2 filter paper. The combined filtrates were concentrated and the compound was purified by column chromatography (5% MeOH/ CH_2_Cl_2_) to afford a crystalline solid in 84% yield.

**^1^H NMR** (300 MHz, CDCl_3_) δ 7.76 (2H, d, *J*=7.2 Hz), 7.60 (2H, d, *J*=6 Hz), 7.42-7.37 (2H, m), 7.33-7.28 (2H, m) 6.25 (1H, br s), 5.6 (1H, d, *J*=7.5 Hz), 5.39 (1H, t, *J*=3.3 Hz), 5.27-5.23 (1H, m), 5.16-5.12 (1H, m), 4.68-4.63 (1H, m), 4.39 (2H, d, *J*=6.6), 4.33-4.05 (5H, m), 3.37-3.25 (2H, m), 2.60 (1H, dd, *J*=15.5, 9.8 Hz), 2.44 (1H, dd, *J*=15.5, 4.2 Hz), 2.11 (3H, s), 2.05 (3H, s), 2.04 (3H, s), 2.02 (3H, s), 1.98-1.86 (1H, m), 1.82-1.69 (1H, m), 1.70-1.54 (2H, m); **^13^C NMR** (100 MHz, CDCl_3_) δ 174.4, 172.4, 170.9, 170.4, 170.0, 169.8, 156.1, 143.6, 141.2, 127.7, 127.0, 125.0, 119.9, 69.1, 68.9, 67.8, 67.7, 66.9, 61.2, 53.4, 50.6, 47.0, 39.1, 34.1, 31.7, 28.6, 22.1, 20.7; IR (thin film): 3367, 1748 cm^-1^; LRMS (ESI): Calcd for C_36_H_43_N_2_O_14_ [M+H] ^+^ 726.3, found 727.0.

**[l-Ornithine(galactose)-glycine-glycine]_4_-glycine (OGG-Gal)**

All polypeptides were prepared by linear solid-phase synthesis using standard Fmoc chemistry. A typical procedure started from Fmoc-glycine Wang resin with loading capacities of ~0.066 mmol/g. The resin was swollen in DMF for 30 minutes. The solvent was then drained and 20% piperidine solution in DMF was added. The solution was allowed to stir for 1 hour, then it was drained, and the resin was washed with three aliquots of DMF. Kaiser and TNBS tests for free amine were then performed. Building block (1.5 equivalents) was premixed with 1.5 equivalents of HBTU in DMF, followed by the addition of 1.5 equivalents of DIPEA in DMF. The reaction was stirred for 30 minutes, transferred to the SPS flask and stirred for 24 hours. The flask was drained and the resin was rinsed three times with DMF. Kaiser and TNBS tests were performed to verify the coupling had reached completion (negative test outcome). The resin was treated with 20% piperdine in DMF solution for 1 hour, the flask was drained and the resin was washed thoroughly with DMF. Kaiser and TNBS tests were performed to ensure the presence of free amine. Then the next successive building block (5 equivalents) to be coupled (commercially available amino acid) was premixed with 5 equivalents of HBTU and 5 equivalents of DIPEA in DMF for 20 minutes. The reaction solution was then transferred to the resin, and the reaction mixture was allowed to stir for 4 hours. The Fmoc deprotection and coupling steps were repeated until twelve amino acid residues were coupled to the resin. To cleave the glycopeptide from the resin, the resin was successively rinsed with DMF, MeOH, and CH_2_Cl_2_, and stirred for 2 hours in 1:1 (v:v) trifluoroacetic acid and CH_2_Cl_2_. The solution was filtered and concentrated, and the product crystallized from diethyl ether to produce a white powder. The glycopolymer was dissolved in a sodium methoxide (pH=10) solution (4:1 sodium methoxide/water) and stirred for 5 hours. The solution was neutralized with IR-120 ion exchange resin, filtered, concentrated to remove MeOH and lyophilized to give the product as a white powder. Purification via HPLC using reverse phase chromatography (C-18 column, liquid phase: 0.1% TFA in acetonitrile/water) was then performed to obtain the desired glyopeptide.

**^1^H NMR** (300 MHz, D_2_O) δ 4.42-4.32 (4H, m), 4.28-4.18 (4H, m), 4.00-3.80 (29H, m), 3.75-3.67 (4H, m), 3.67-3.47 (14H, m), 3.20-3.05 (8H, m), 2.62-2.42 (8H, m), 1.85-1.70 (5H, m), 1.70-1.57 (4H, m), 1.57-1.37 (9H, m); **^13^C NMR** (75 MHz, D_2_O) δ 175.0, 174.9, 174.1, 173.9, 173.6, 172.3, 172.3, 172.2, 172.0, 171.9, 171.8, 170.7, 163.6, 163.1, 73.3, 72.8, 70.0, 69.1, 67.9, 61.2, 54.1, 53.2, 42.9, 42.7, 41.5, 41.4, 39.1, 32.7, 28.4, 25.2; LRMS (MALDI-TOF): Calcd for C_70_N_117_N_17_O_38_ [M+H]^+^ 1805.8, found 1805.3.

## Synthesis of *N*-octyl-d-gluconamide (NOGlc)

To a solution of d-gluconic acid-δ-lactone (1.4 g, 7.86 mmol) in MeOH (30 mL) was added *n*-octylamine (1.3 mL, 7.86 mmol). The mixture was refluxed for 1 hour then cooled in an ice bath. The precipitate was filtered off and washed with cold MeOH to afford **NOGlc** as a white powder (1.45 g, 60%). Characterization data is consistent with that previously reported in the literature.

**^1^H NMR** (500 MHz, DMSO-*d_6_*) δ 7.59, (t, *J* = 6.0 Hz, 1H), 5.34 (d, *J* = 5.1 Hz, 1H), 4.53 (t, *J* = 4.8 Hz, 1H), 4.47 (d, *J* = 5.1 Hz, 1H), 4.39 (d, *J* = 7.2 Hz, 1H), 4.33 (d, *J* = 5.8 Hz, 1H), 3.97 (dd, *J* = 4.9, 3.8 Hz, 1H), 3.89 (ddd, *J* = 7.2, 3.7, 2.2 Hz, 1H), 3.57 (m, 1H), 3.46 (m, 2H), 3.37 (m, 1H), 3.06 (m, 2H), 1.40 (quint, *J* = 6.6 Hz, 2H), 1.31-1.18 (m, 10H), 0.86 (t, *J* = 6.7 Hz, 3H). **^13^C NMR** (125 MHz, DMSO-*d_6_*) δ 172.2, 73.6, 72.4, 71.5, 70.1, 63.4, 38.3, 31.3, 29.2, 28.8, 28.7, 26.4, 22.1, 14.0. LRMS (ESI): *m/z* calcd. for C_14_H_30_NO_6_ [M+H]^+^ 308.2; found 308.3.

## Synthesis of *N*-octyl-d-galactonamide (NOGal)

A solution of calcium galactonate (200 mg, 0.46 mmol) in methanol (2 mL) was cooled to 0 °C and SOCl_2_ (70 **μ**L, 0.93 mmol) was added dropwise. The solution was slowly warmed to room temperature and was stirred overnight. The mixture was then evaporated and dried *in vacuo* to give 200 mg of a white powder. This powder was then dissolved in methanol (2 mL) and *n*-octylamine (230 **μ**L, 1.44 mmol) was added. The mixture was refluxed for 2 hours then cooled in an ice bath. The precipitate was filtered off and washed with cold methanol to afford **NOGal** as a white powder (112 mg, 38%). Characterization data is consistent with that previously reported in the literature.

**^1^H NMR** (500 MHz, DMSO-*d_6_*) δ 7.53, (t, *J* = 6.0 Hz, 1H), 5.07 (d, *J* = 7.2 Hz, 1H), 4.44 (t, *J* = 5.6 Hz, 1H), 4.28 (d, *J* = 8.1 Hz, 1H), 4.17 (d, *J* = 6.6 Hz, 1H), 4.12 (d, *J* = 7.5 Hz, 1H), 4.08 (d, *J* = 8.1 Hz, 1H), 3.78 (t, *J* = 8.7 Hz, 1H), 3.69 (q, *J* = 5.6 Hz, 1H), 3.45-3.37 (m, 3H), 3.07 (m, 2H), 1.40 (m, 2H), 1.31-1.18 (m, 10H), 0.86 (t, *J* = 6.6 Hz, 3H). **^13^C NMR** (125 MHz, DMSO-*d_6_*) δ 173.3, 70.9, 70.7, 69.8, 69.1, 63.2, 38.3, 31.3, 29.3, 28.8, 28.7, 26.4, 22.1, 14.0. LRMS (ESI): *m/z* calcd. for C_14_H_30_NaNO_6_ [M+Na]^+^ 330.2; found 330.2.

# Spectroscopic Data

**2,3,4,6-tetra-*O*-acetyl-α-d-galactopyranosyl bromide (2)**


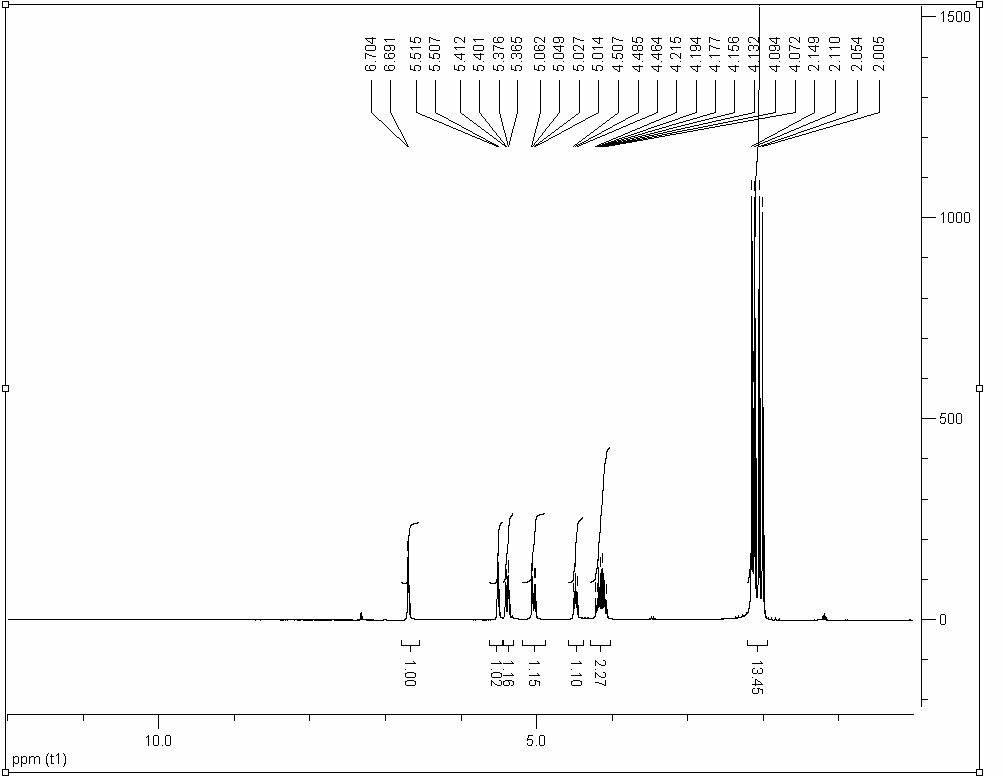

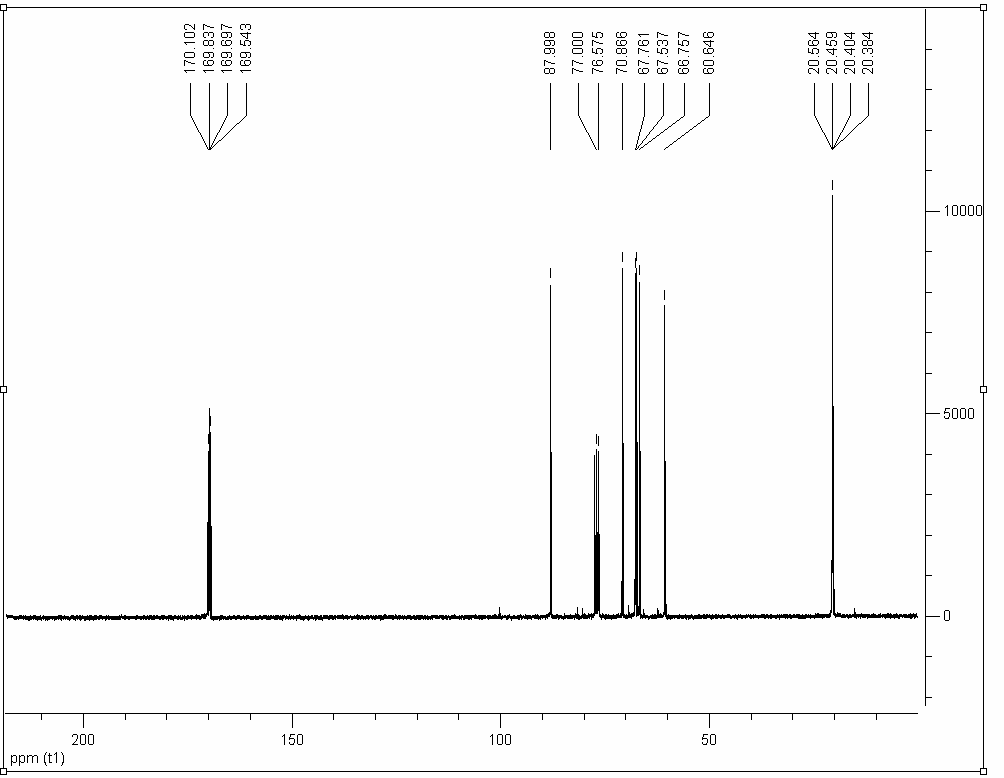


**Allyl 2,3,4,6-tetra-*O*-acetyl-α-d-galactopyranoside (3)**


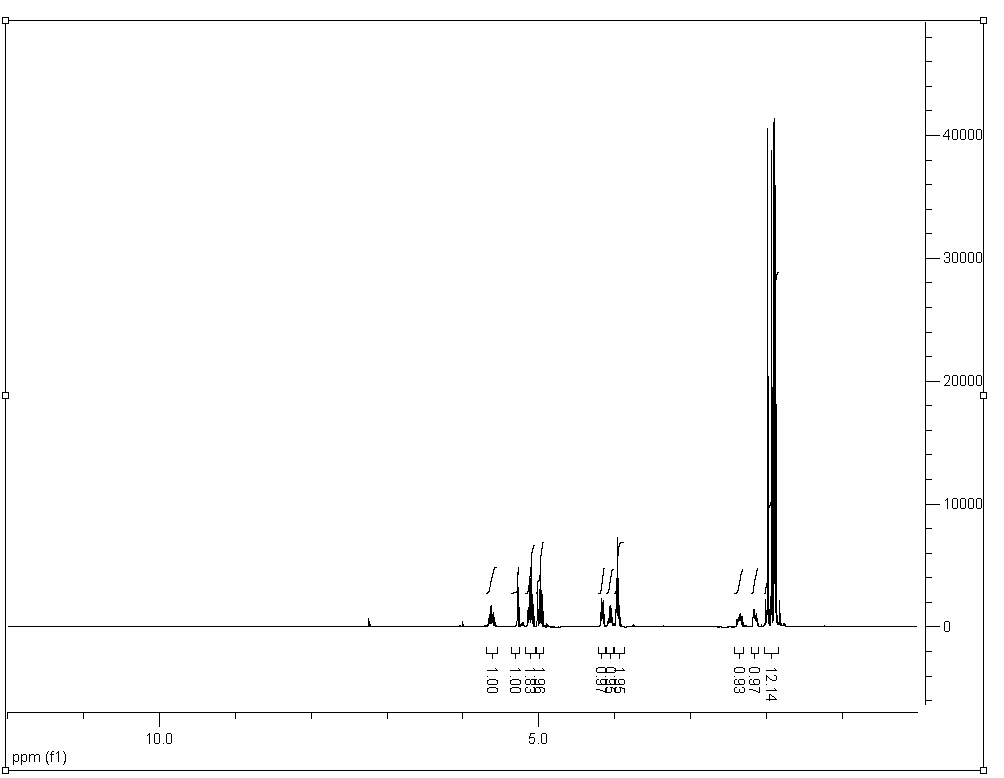


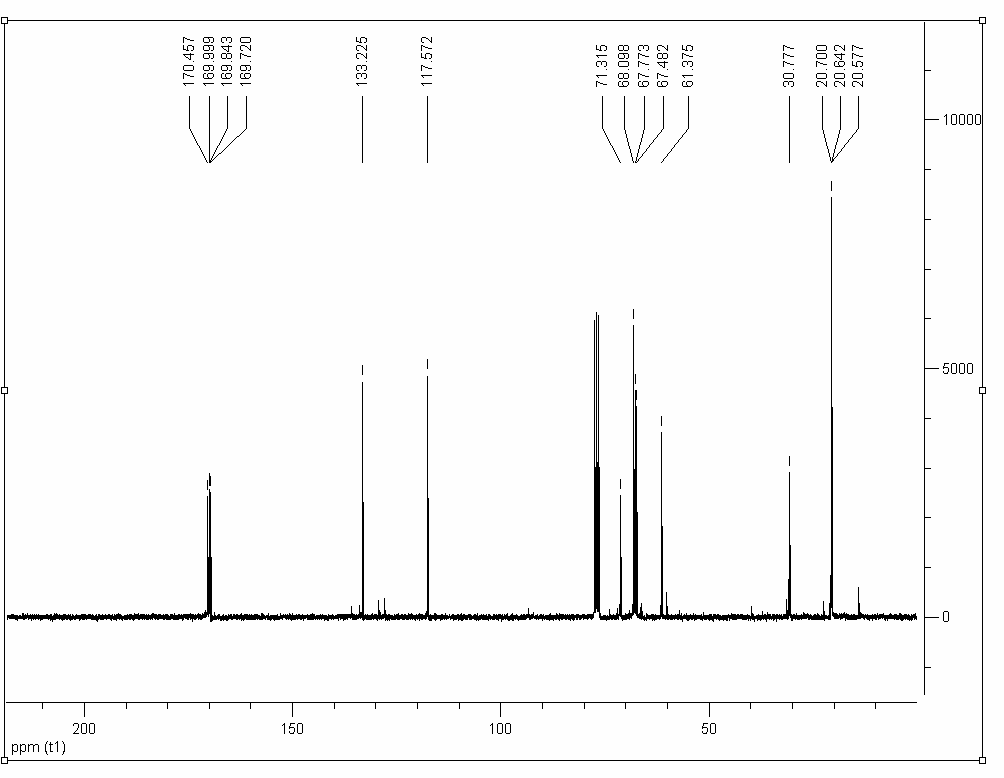


**2,3,4,6-tetra-*O*-acetyl-α-d-galactopyranosyl acid (4)**


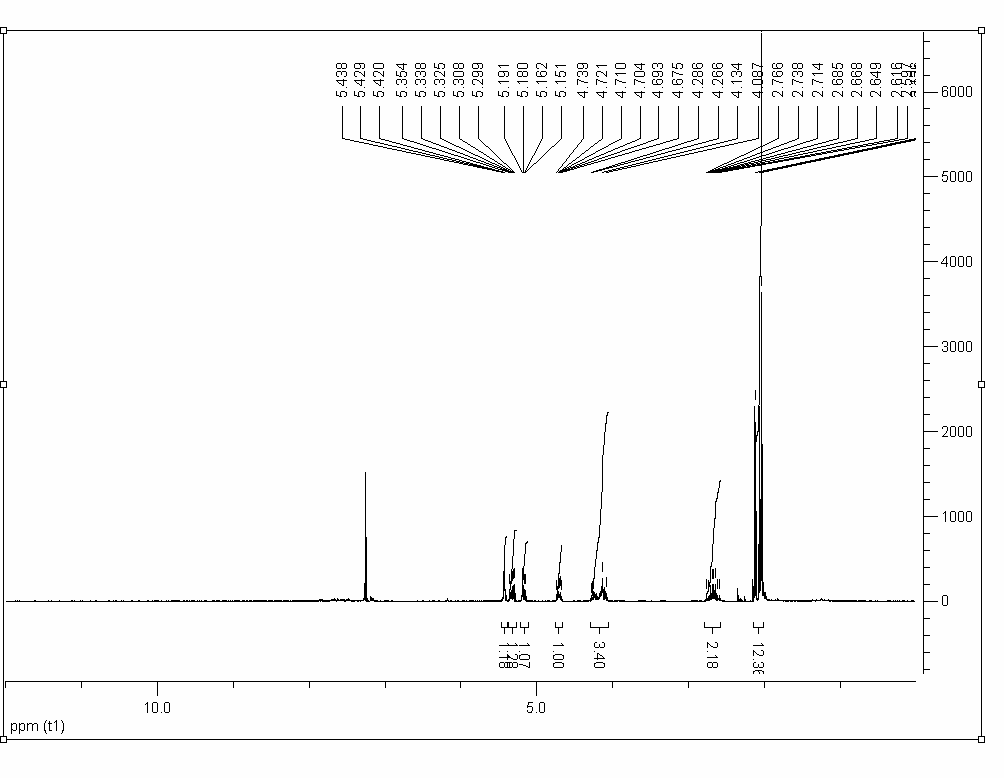


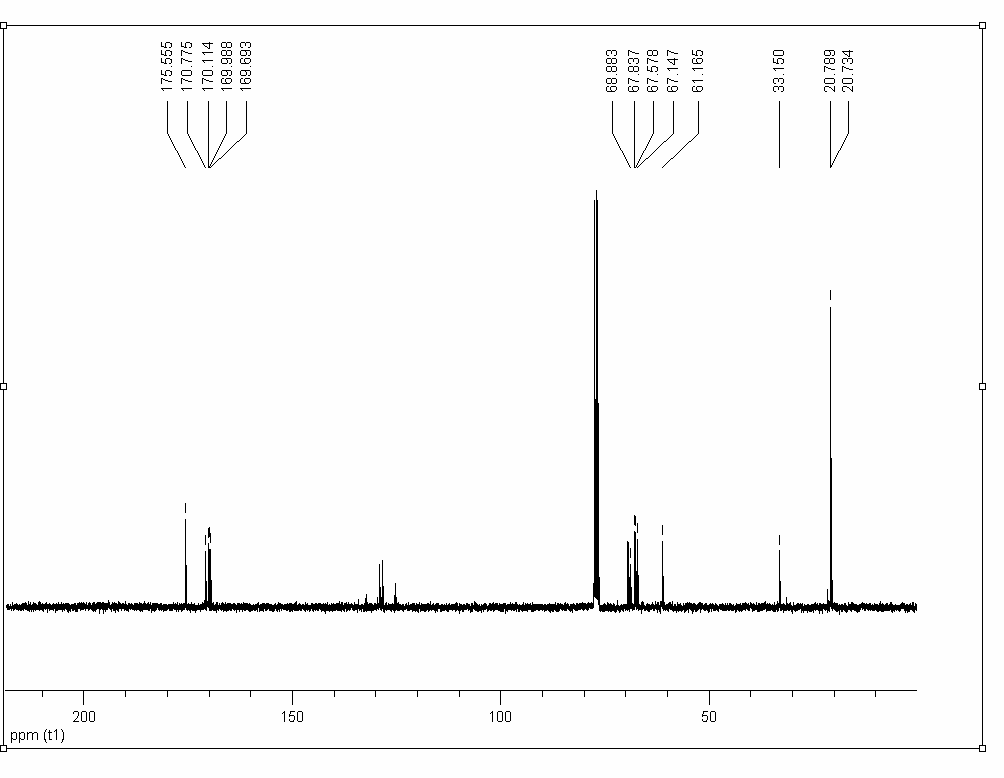


**Benzyl Fluorenylmethoxycarbonyl-l-ornithine-1-(2,3,4,6-tetra-*O*-acetyl-α-d-galacto-pyranoside) (6)**


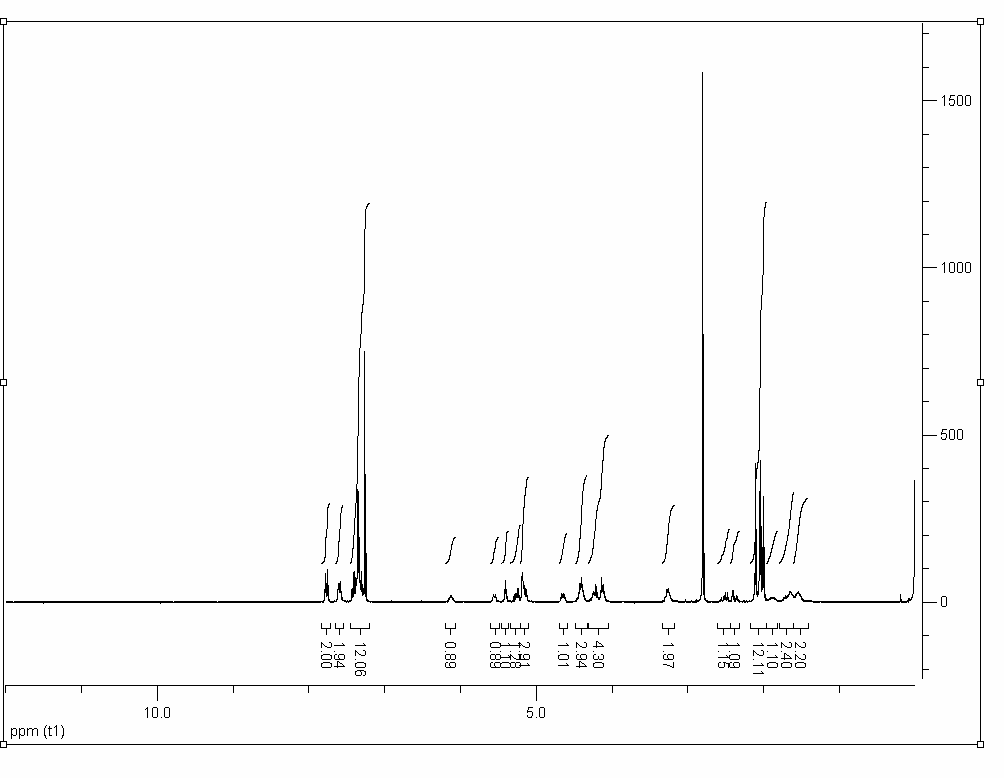


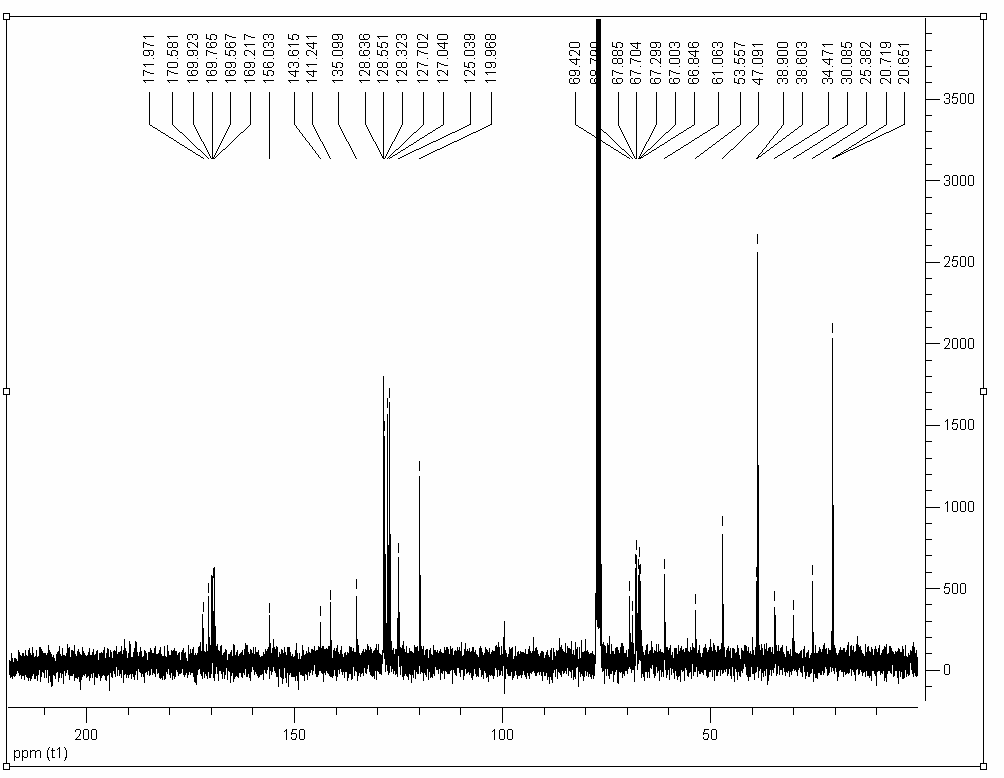


**Fluorenylmethoxycarbonyl-l-ornithine-1-(2,3,4,6-tetra-*O*-acetyl-α-d-galacto-pyranoside) (7)**


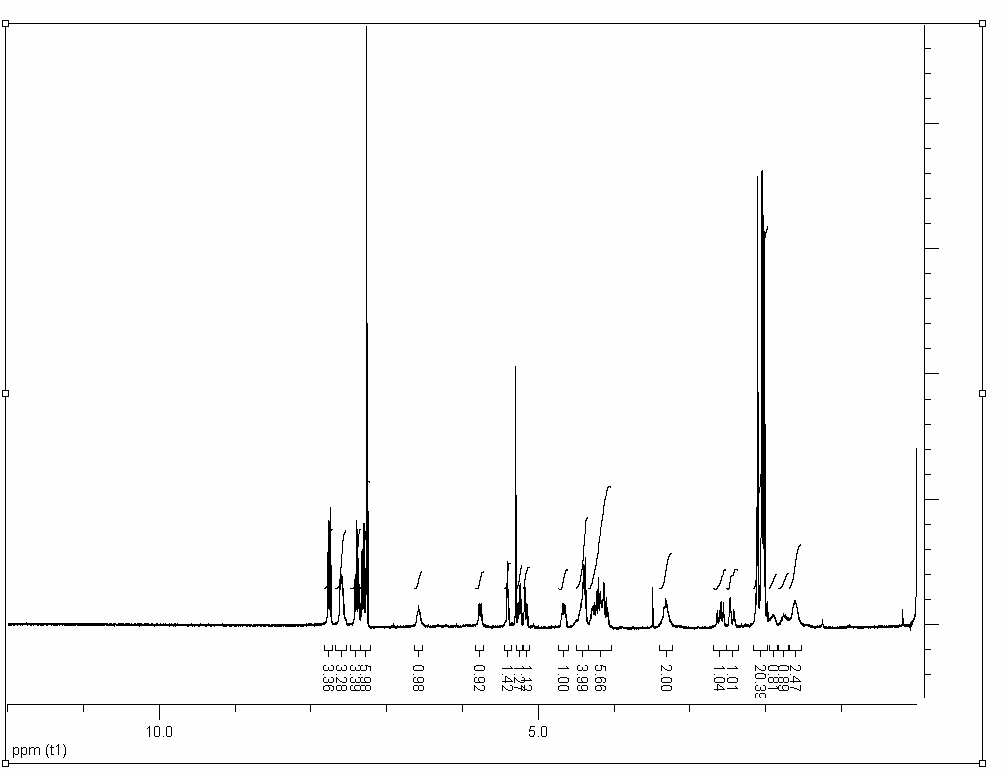


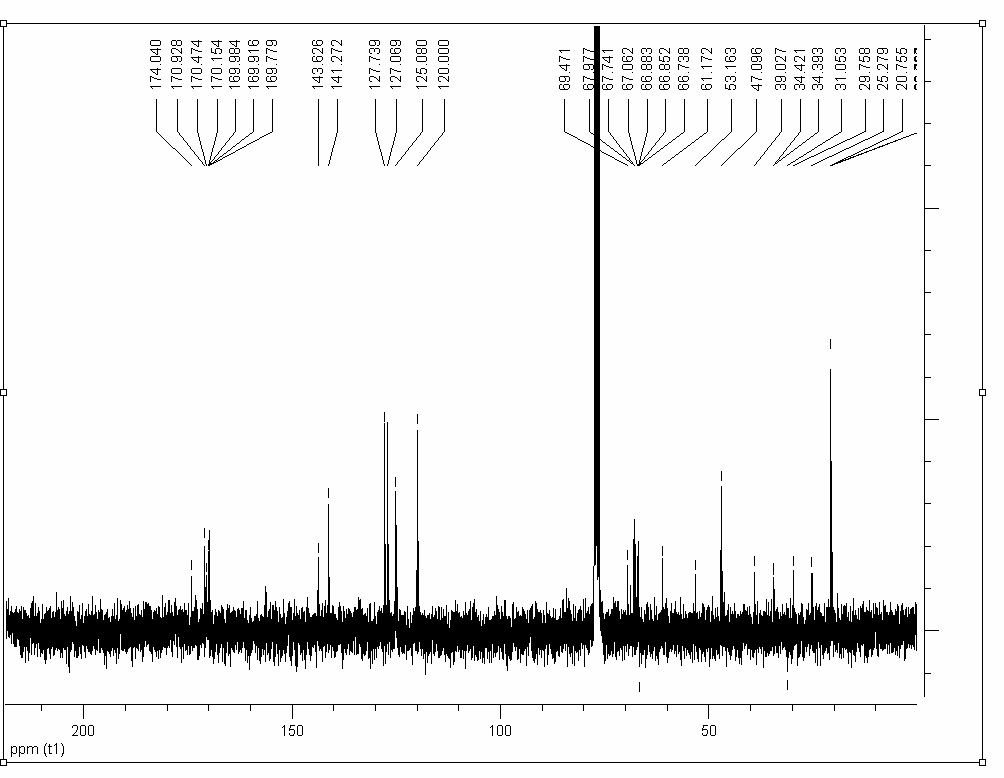


**[l-Ornithine(galactose)-glycine-glycine]_4_ -glycine (OGG-Gal)**


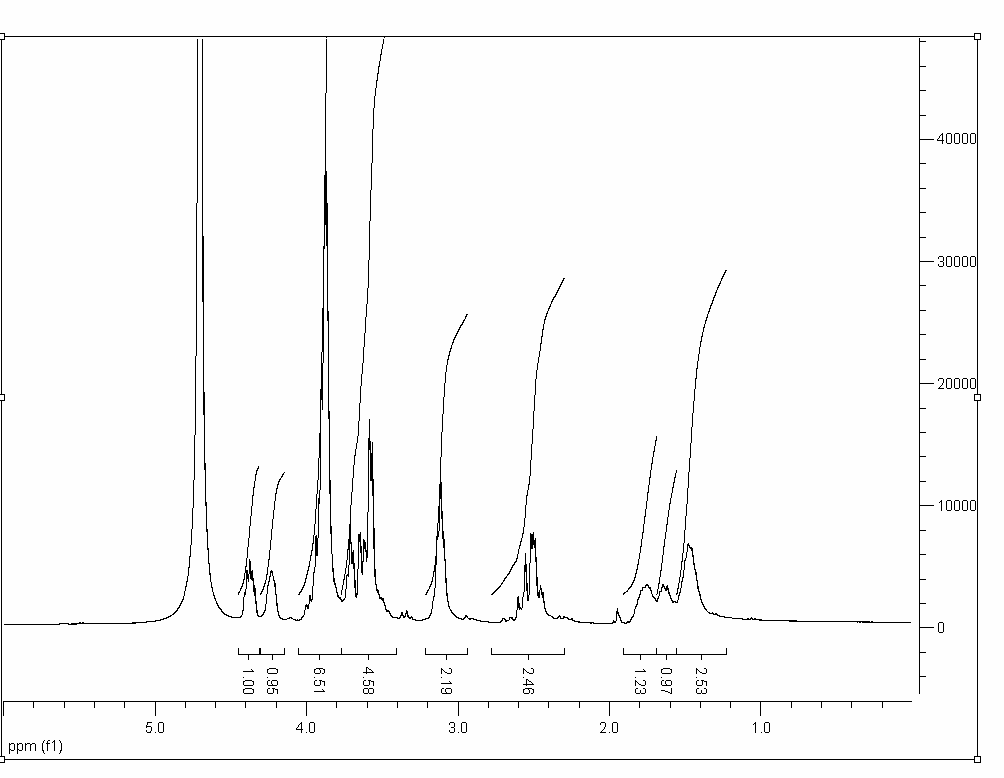


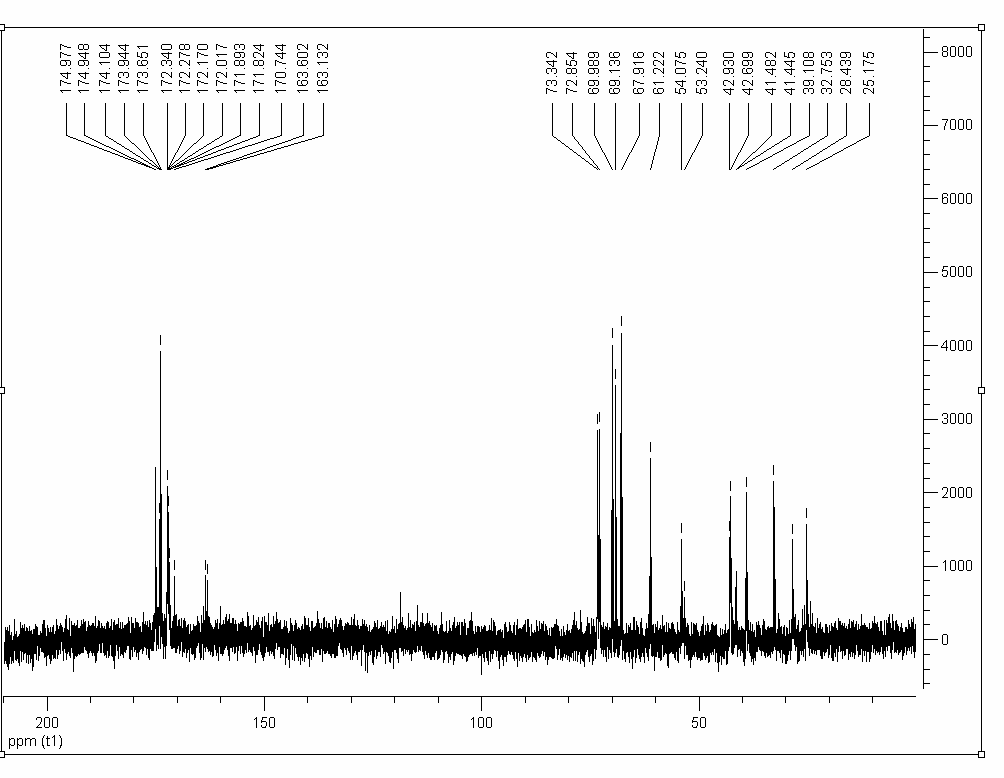


***N*-octyl-d-gluconamide (NOGlc)**

***N*-octyl-d-galactonamide (NOGal)**

# References

1 C. A. Knight, J. Hallett, A. L. DeVries, *Cryobiology* **1988**, *25*, 55-60.

2 J. Jackman, M. Noestheden, D. Moffat, J. P. Pezacki, S. Findlay, R. N. Ben, *Biochem. Bioph. Res. Co.* **2007**, *354*, 340-344.

3 G. B. Fields, C. g. Fields, *J. Am. Chem. Soc.* **1991**, *113*, 4202-4207.

4 a) S. Svenson, B. Kirste, J.-H. Fuhrhop, *J. Am. Chem. Soc.* **1994**, *116*, 11969-11975; b) S. Svenson, A. Schafer, J.-H. Fuhrhop, *J. Chem. Soc. Perk. T. 2* **1994**, 1023-1028.
